# A Novel High Sensitivity Type II Collagen Blood-Based Biomarker, PRO-C2, for Assessment of Cartilage Formation

**DOI:** 10.3390/ijms19113485

**Published:** 2018-11-06

**Authors:** Yunyun Luo, Yi He, Ditte Reker, Natasja Stæhr Gudmann, Kim Henriksen, Ole Simonsen, Christoph Ladel, Martin Michaelis, Ali Mobasheri, Morten Karsdal, Anne-Christine Bay-Jensen

**Affiliations:** 1 Department of Rheumatology, Nordic Bioscience, Biomarkers and Research, 2730 Herlev, Denmark; yhe@nordicbio.com (Y.H.); dre@nordicbio.com (D.R.); nsg@nordicbio.com (N.S.G.); kh@nordicbio.com (K.H.); mk@nordicbio.com (M.K.); acbj@nordicbio.com (A.-C.B.-J.); 2 Faculty of Health and Medical Sciences, University of Copenhagen, 2200 København, Denmark; 3 Department Orthopedic Surgery, Aalborg University Hospital, 9000 Aalborg, Denmark; ohs@rn.dk; 4 Merck KGaA, 64293 Darmstadt, Germany; christoph.ladel@merckgroup.com (C.L.); martin.michaelis@merckgroup.com (M.M.); 5 D-BOARD EU Consortium for Biomarker Discovery, Faculty of Health and Medical Sciences, University of Surrey, Guildford GU2 7AL, Surrey, UK; a.mobasheri@surrey.ac.uk; 6 Arthritis Research UK Centre for Sport, Exercise and Osteoarthritis, Queen’s Medical Centre, Nottingham NG7 2UH, Nottinghamshire, UK; 7 Department of Regenerative Medicine, State Research Institute Centre for Innovative Medicine, LT-01102 Vilnius, Lithuania

**Keywords:** osteoarthritis, cartilage formation, PIIANP, type II collagen, PIIBNP

## Abstract

N-terminal propeptide of type II collagen (PIINP) is a biomarker reflecting cartilage formation. PIINP exists in two main splice variants termed as type IIA and type IIB collagen NH_2_-propeptide (PIIANP, PIIBNP). PIIANP has been widely recognized as a cartilage formation biomarker. However, the utility of PIIBNP as a marker in preclinical and clinical settings has not been fully investigated yet. In this study, we aimed to characterize an antibody targeting human PIIBNP and to develop an immunoassay assessing type II collagen synthesis in human blood samples. A high sensitivity electrochemiluminescence immunoassay, hsPRO-C2, was developed using a well-characterized antibody against human PIIBNP. Human cartilage explants from replaced osteoarthritis knees were cultured for ten weeks in the presence of growth factors, insulin-like growth factor 1 (IGF-1) or recombinant human fibroblast growth factor 18 (rhFGF-18). The culture medium was changed every seven days, and levels of PIIBNP, PIIANP, and matrix metalloproteinase 9-mediated degradation of type II collagen (C2M) were analyzed herein. Serum samples from a cross-sectional knee osteoarthritis cohort, as well as pediatric and rheumatoid arthritis samples, were assayed for PIIBNP and PIIANP. Western blot showed that the antibody recognized PIIBNP either as a free fragment or attached to the main molecule. Immunohistochemistry demonstrated that PIIBNP was predominately located in the extracellular matrix of the superficial and deep zones and chondrocytes in both normal and osteoarthritic articular cartilage. In addition, the hsPRO-C2 immunoassay exhibits acceptable technical performances. In the human cartilage explants model, levels of PIIBNP, but not PIIANP and C2M, were increased (2 to 7-fold) time-dependently in response to IGF-1. Moreover, there was no significant correlation between PIIBNP and PIIANP levels when measured in knee osteoarthritis, rheumatoid arthritis, and pediatric serum samples. Serum PIIBNP was significantly higher in controls (KL0/1) compared to OA groups (KL2/3/4, *p* = 0.012). The hsPRO-C2 assay shows completely different biological and clinical patterns than PIIANP ELISA, suggesting that it may be a promising biomarker of cartilage formation.

## 1. Introduction

Osteoarthritis (OA) is generally characterized by a slowly progressive degeneration of articular cartilage [1,2]. The extracellular matrix (ECM) in articular cartilage consists primarily of proteoglycans and type II collagen [3,4]. In healthy cartilage, the ECM is characterized by a low turnover of collagens, whereas in OA, the homeostasis is disrupted, leading to an imbalance between cartilage formation and degradation [5]. In other words, a net loss of cartilage results in diseased cartilage [6,7].

Currently, there are no disease-modifying OA drugs (DMOADs) available. Those in development are either preventing cartilage breakdown (anti-catabolic pathways) or targeting cartilage formation (anabolic pathways). According to the list of DMOADs in past and present clinical development registered in clinicaltrials.gov, the majority of these trials are based on anti-catabolic or anti-inflammatory approaches [8]. Anabolic compounds under evaluation for cartilage regeneration or repair include but are not limited to insulin growth factor 1 (IGF-1) and recombinant human fibroblast growth factor 18 (rhFGF-18 or sprifermin). The ability of IGF-1 to influence chondrocyte proliferation and ECM turnover is well established [9,10,11]. Sprifermin has been shown to enhance cartilage synthesis in the settings of in vitro [12], ex vivo [13], and clinical trials [14,15,16]. The reason that there are few anabolic trials may in part be due to the lack of valid tools measuring the anabolic processes. Presently, the measurement of cartilage formation relies on magnetic resonance imaging (MRI) and radiographic techniques, which is a sensitive means but only observes the outcome after following up on the patient for a long time. Therefore, there is an unmet need in DMOAD development, where non-invasive biomarkers of cartilage formation can provide an early indication of drug efficacy.

As the major matrix protein in articular cartilage, type II collagen is synthesized as a procollagen consisting of propeptides at both the C and N-terminus (PIICP and PIINP). PIINP exists in two splice variants termed type IIA collagen NH_2_-propeptide (PIIANP) and type IIB collagen NH_2_-propeptide (PIIBNP) in consequence of alternative RNA splicing [17,18]. PIIBNP differs from PIIANP with the absence of exon 2 encoding a cysteine-rich globular domain [19]. PIIANP is synthesized by chondroprogenitor cells and is usually restricted to embryogenesis and fracture healing, and it may also be re-expressed in osteoarthritic cartilage and osteophytes [5,20,21,22]; it is also thought to represent a dedifferentiated version of type II collagen [23]. On the other hand, PIIBNP is considered to be the only procollagen splice variant that is mainly expressed during type II collagen formation in healthy adult cartilage [21]. These propeptides are cleaved from the collagen triple helix during secretion by specific proteinases (e.g., matrix metalloproteinases, N-proteinase, and C-proteinase) before being released into the circulation [21,24,25,26]. Therefore, the amount of free propeptide directly correlates with type II collagen synthesis, a relationship analogous to that of the carboxy-terminal proinsulin peptide and endogenously produced insulin [5,23,27].

Quantitative detection of type II collagen synthesis was previously reported by Rousseau et al., who described a competitive immunoassay for PIIANP using polyclonal antibodies [27]. We have previously reported on the development of a PIIBNP enzyme-linked immunosorbent assay (ELISA) reflecting cartilage formation [28]. However, the lack of sensitivity for measuring the fragment in clinical samples hampered its application [28]. Thus, the aim of the present study was to characterize a monoclonal antibody against human PIIBNP as well as to enable an assessment of type IIB collagen synthesis in human serum by switching the assay onto a more sensitive platform. Additionally, we made a comparison of PIIANP and the new PIIBNP (hereafter called hsPRO-C2) assay.

## 2. Results

### 2.1. Characterization of a Monoclonal Antibody

#### 2.1.1. Binding Specificity

**Figure 1:**
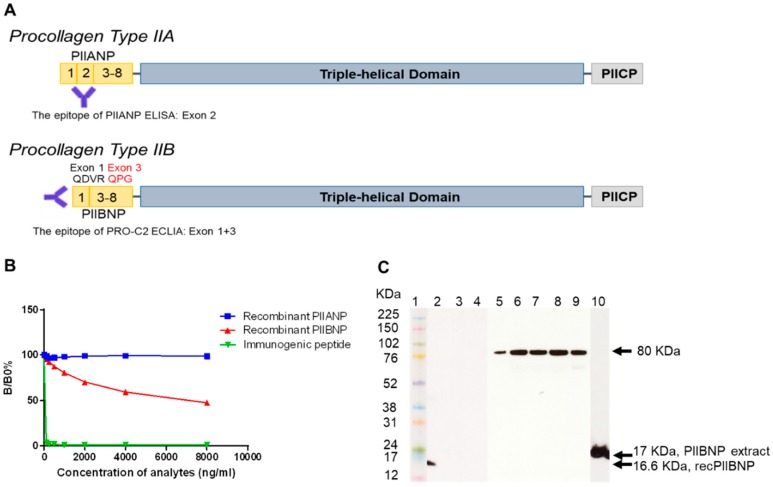
(**A**) Schematic illustration of type IIA collagen NH_2_-propeptide (PIIANP) and type IIB collagen NH_2_-propeptide (PIIBNP). Type II procollagen is synthesized in two splice forms, type IIA and IIB, as the result of alternative splicing of exon 2 in the N-terminal propeptide region. The polyclonal antibody employed in the PIIANP ELISA recognizes the region encoded by exon 2. The monoclonal antibody utilized in the high sensitivity type IIB collagen NH_2_-propeptide (hsPRO-C2) electrochemiluminescence immunoassay (ECLIA) only recognizes the N-terminus of PIIBNP encoded by the junction of exon 1 and 3 (QDVRQPG). (**B**) Antibody specificity test. The signal of the NB443-3-2-1 antibody was displaced by increasing the concentration of immunogenic peptide (QDVRQPGPKG) and PIIBNP recombinant protein, but not by PIIANP recombinant protein. (**C**) Western blot detection of the PIIBNP protein. Marker molecular weights are indicated in lane 1. Recombinant human PIIBNP (lane 2), recombinant human PIIANP (lane 3), pooled synovial fluid from knee osteoarthritis (OA) patients (lane 5), pooled sera from healthy adults (lane 6), OA patients (lane 7), rheumatoid arthritis (RA) patients (lane 8), pooled human amniotic fluid (lane 9), and Pronase E-extracted normal human articular cartilage (lane 10) were incubated with NB443-3-2-1. Recombinant human PIIBNP (lane 4) was incubated with normal mouse IgG as a negative control. The positive control is recombinant human PIIBNP showing the molecular mass of the PIIBNP to be 16.6 KDa.

The monoclonal antibody, NB443-3-2-1, was developed against the exon 1/3 junction encoded sequence of PIIBNP (QDVRQPGPKG), which is different from PIIANP (Figure 1A). The signal of NB443-3-2-1 (IgG1, κ) was displaced by increasing the concentration of immunogenic peptide (QDVRQPGPKG) and recombinant PIIBNP but not by PIIANP (Figure 1B).

#### 2.1.2. Western Blot

The NB443-3-2-1 antibody detected a 16.6 KDa band for recombinant PIINBP (lane 2, Figure 1C), which coincides with the theoretical molecular weight (MW) of PIIBNP. No bands were detected for the recombinant PIIANP or the negative control (lane 3 and 4, respectively). A band of 80 KDa was clearly detectable in synovial fluid from knee OA patients (lane 5), sera from healthy adults (lane 6) and OA patients (lane 7), RA patients (lane 8), and human ammonic fluid (lane 9). A 17 KDa band was detected from the normal human articular extract (lane 10), which migrated slowly than the recombinant PIIBNP due to the hydroxylation of proline residues as Wang et al reported [29].

### 2.2. Immunolocalization of PIIBNP in Human Articular Cartilage

**Figure 2:**
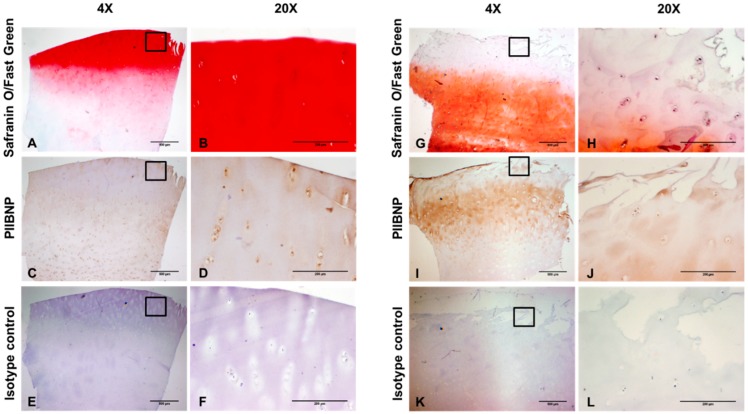
Immunolocalization of PIIBNP in normal (**A**–**F**) and OA (**G**–**L**) human articular cartilage. (**A**,**G**) Safranin O/Fast green histology staining, 4× magnification; (**B**,**H**) Higher magnification (20×) images of the squares in panel A+G are shown; (**C**,**I**) Immunolabeling with an anti-PIIBNP antibody, 4× magnification; (**D**,**J**) Higher magnification (20×) images of the squares in panel C+I are shown; (**E**,**K**) IgG1 isotype control, 4× magnification; (**F**,**L**) Higher magnification (20×) images of the squares in panel E+K are shown. Scale bar: 500 µm (**A**,**C**,**E**,**G**,**I**,**K**), 200 µm (**B**,**D**,**F**,**H**,**J**,**L**).

In the normal human articular cartilage, safranin O/fast green staining was intense red on the surface, indicating high proteoglycan contents (Figure 2A,B). PIIBNP was moderately stained in the cytoplasm of chondrocytes. Apparent staining of PIIBNP was also seen in the extracellular matrix of the superficial, middle, and deep zones (Figure 2C,D). By contrast, Safranin O/fast green staining was weak orange in the human OA cartilage, where the superficial zone was fibrillated and lost the majority of proteoglycan (Figure 2G,H). Surprisingly, there was moderate staining of PIIBNP in the superficial zone but strong staining in the middle zone (Figure 2I,J). No specific staining was found in the section incubated with normal mouse IgG1 (Figure 2E,F,K,L).

### 2.3. Development and Validation of hsPRO-C2 ElectroChemiLuminescence ImmunoAssay (ECLIA)

#### 2.3.1. Technical Performance of hsPRO-C2 ECLIA

A competitive ECLIA was developed by applying the NB443-3-2-1 antibody. Technical performance of this ECLIA was summarized in Table 1. The intra-assay coefficient of variation (CV) was 5.4% and the inter-assay CV was 5.5%. The measurement range was 1.0–38.5 ng/mL. Spiking and dilution recovery tested in human serum was 100 ± 20% within the measurement range of the assay (Supplementary Figure S3A). The effect of different anticoagulants was tested and there were significant correlations between values in human serum and three kinds of plasmas (*r* = 0.92 Ethylenediaminetetraacetic acid plasma, *r* = 0.90 citrate plasma, *r* = 0.91 heparinized plasma, Supplementary Figure S3B).

#### 2.3.2. Induction of PIIANP and PIIBNP Release in Human OA Cartilage Explants by Growth Factors

**Figure 3:**
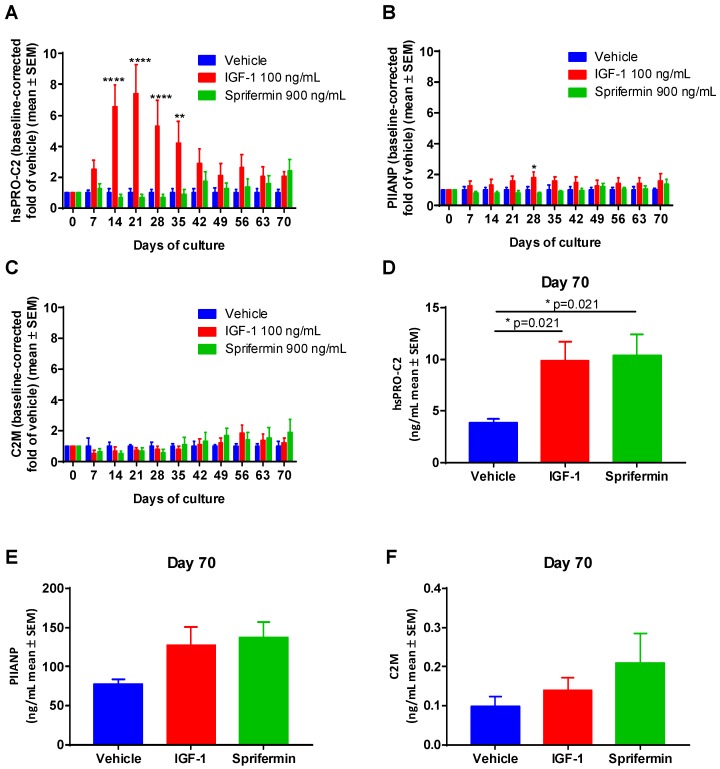
hsPRO-C2 (**A**,**D**), PIIANP (**B**,**E**) and C2M (**C**,**F**) measured in the supernatant of human articular cartilage explants cultured for ten weeks in the presence of growth factors (IGF-1 and sprifermin) compared to the vehicle group. The values in panels (**A**–**C**) and panels (**D**–**F**) were compared with ordinary two-way analysis of variance (ANOVA) and one-way ANOVA test (not repeated measures) respectively. Asterisks indicate the following: * *p* < 0.05, ** *p* < 0.01, **** *p* < 0.0001.

To investigate if PIIANP and PIIBNP secretions were affected by cartilage anabolic growth factors, human OA explants were treated by sprifermin and IGF-1 respectively. IGF-1 significantly increased the levels of PIIBNP during the early phase and mid-phase of the culture period compared to the vehicle group (Figure 3A). Sprifermin stimulated PIIBNP secretion in the later phase as also described by Reker et al. [13], particularly showing a significant increase in PIIBNP at day 70 in comparison to the vehicle group (Figure 3D). In contrast, PIIANP levels were not induced by such treatments (Figure 3B,E) although IGF-1 resulted in elevated PIIANP levels at day 28. Meanwhile, the concentrations of type II collagen degradation marker (C2M) in IGF-1 and sprifermin groups were not significantly changed during the entire culture period in comparison to the vehicle (Figure 3C,F). The metabolic activities of explants remained during the whole period of culture by measuring alamarblue (Supplementary Figure S3). To ensure the chondrocytes were not dedifferentiated, type I collagen levels were investigated by measuring type I collagen NH_2_-propeptide (PINP) at different time points, which showed nothing.

#### 2.3.3. hsPRO-C2 Concentration in Healthy Subjects and Knee OA Patients

**Figure 4:**
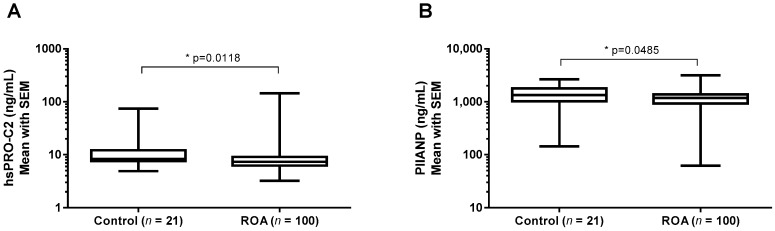
Serum type IIB collagen NH_2_-propeptide (PIIBNP) (**A**) and type IIA collagen NH_2_-propeptide (PIIANP) (**B**) were determined in healthy subjects and osteoarthritic patients. Control group: Kellgren-Lawrence (KL) = 0 and 1, *n* = 22; Radiographic osteoarthritis (ROA) group: KL = 2, 3 and 4, *n* = 123. Black horizontal lines are means, and error bars are standard error of the mean (SEM). The nonparametric Mann–Whitney test was used to compare each group. * *p* < 0.05.

Subjects were divided into two groups according to their KL grades. There was no marked difference in the sex and age distribution across the groups (Table 2). Body mass index (BMI) and visual analog scale (VAS) were significantly higher in knee OA compared to the control group (*p* = 0.021, *p* = 0.043, Table 2). To investigate whether these markers had any clinical relevance, serum samples from 123 knee OA patients in different stages of the disease and healthy controls were measured in the PIIBNP and PIIANP assays. The mean PIIBNP level was significantly lower in the knee OA group than the control group (*p* = 0.012, Figure 4A) after adjusting for age, sex, and BMI. Mean PIIANP level demonstrated similar results, but there was a less significant difference between the groups (*p* = 0.049, Figure 4B). There was no correlation between PIIBNP and PIIANP observed when assessed in the available serum samples (Table 3).

## 3. Discussion

There are several biomarkers available in the field of OA which assess cartilage degradation in the serum or urine of patients; however, there are very few that detect the formation of cartilage. It would be desirable to be able to measure both the degradation and the formation of cartilage to provide insight into the turnover and remodeling of cartilage during the pathogenesis of the disease or in response to a DMOAD. We used the monoclonal antibody from previously published PRO-C2 ELISA for the development of ECLIA [28]. In addition, we looked deeper into the characterization of the monoclonal antibody to provide better insight into the specificity of the assay.

The targeted epitope QDVRQPGPKG was human type IIB collagen-specific. As previously reported, the monoclonal antibody had no cross-reactivity with the synthetically generated form of human PIIANP sequence (QDVQEAGSCV) and the mouse or rat PIIBNP sequence (QDARKLGPKG) [28]. The specificity of the antibody to PIIBNP was further confirmed by Western blot using recombinant PIIBNP, recombinant PIIANP, and human articular cartilage tissue. NB443-3-2-1 detected one band with the MW being 17 KDa, which corresponds to the native PIIBNP and is in agreement with previous findings [29]. However, interestingly, only one band of 80 KDa was observed in human synovial fluid from patients with knee OA, serum from healthy adults, and patients with RA or OA. Similarly, Rousseau et al. discovered that the MW of PIIANP present in human sera and synovial fluid was also around 80 KDa, which is much larger than the theoretical MW of native PIIANP [27]. We speculate that the bands of 80 KDa found in both situations likely stem from the multiple N-terminal propeptides, which include triple helical regions bound to each other in the N-terminal telopeptide region by cross-linking. Another possible reason could be that the N-terminal propeptide, if present in sera, is somehow masked or cross-linked with other collagen molecules, e.g., type III, IX, or XI collagen due to its small size [27]. In immunohistochemistry, we demonstrated that PIIBNP was predominately located in the extracellular matrix of the superficial and deep zones and chondrocytes in both normal and osteoarthritic articular cartilage, which is in agreement with the report by Aubert-Foucher et al. [30]. Interestingly, the osteoarthritic articular cartilage showed intense staining compared to the normal cartilage, which may due to the unmatched age and sex of both samples. As we observed in Figure 4, some OA patients have relatively high PRO-C2 levels, which can also explain the immunohistological difference.

Here, we described a second-generation PIIBNP quantification assay when converting from the traditional ELISA format to the ECLIA format on the Mesoscale Discovery (MSD) platform. We have shown how the use of a new platform has resulted in a seven-fold increased sensitivity in the ECLIA assay (LLOD: 1 ng/mL vs. 0.13 ng/mL, Table 1), allowing for quantification also in serum and plasma. There was a strong correlation (*r* = 0.9414, Figure S2C) between absolute values of PRO-C2 using the two platforms, although the new assay against the old assay showed a three-fold increment of concentration values. Thus, the relative differences in PRO-C2 concentration between samples were the same regardless of which platform was used. We observed that IGF-1 and sprifermin demonstrated the ability to prompt type IIB collagen formation assessed by hsPRO-C2 in the human cartilage explants (HEX) ex-vivo study. Nevertheless, the PIIANP levels were not affected significantly. As the degradation of type II collagen (C2M) was not significantly affected by IGF-1 and sprifermin compared to the vehicle group, the net type IIB collagen content was increased after induction. Previously, we have shown that PIIBNP secretion was also increased in bovine articular explants stimulated with TGF-β1. Meanwhile, the PIIBNP levels were decreased significantly when the bovine explants were treated by catabolic cytokines, namely a combination of Oncostatin M and TNF-α [28]. Considered together, the hsPRO-C2 assay is indicative of type II collagen formation rather than degradation. The levels of PIIBNP are significantly higher in healthy controls in comparison to knee OA patients, suggesting that it is to some degree associated with disease severity. The PIIANP concentrations are also reduced in knee OA patients, which is in accordance with a previous report [27]. hsPRO-C2 ECLIA was not associated with the commercially available PIIANP assay, which to some extent reflects the different structures and functions of both N-terminal propeptides.

The development of simple and reliable non-invasive biomarkers of OA, especially the identification of novel biomarkers that are able to accurately and relatively quickly assess the efficacy of therapy, is an important goal in clinical rheumatology. It will facilitate the design and evaluation of clinical trials on DMOADs [31]. In the past decade, many discoveries of cartilage formation biomarkers have been reported—e.g., PIINP, PIIANP, and PIICP (also called CPII or chondrocalcin). Olsen et al. applied the F7504 antibody that recognizes the neo-epitope GPQGPAGEQGPRGDR in the exon 5-6 of PIINP and developed a PIINP assay [9]. Nevertheless, this PIINP assay detects a linear amino-acids sequence that is present in both PIIANP and PIIBNP variants, thereby provoking specificity concerns. On the other hand, the PIIANP assay only detects the exon 2 sequence in the N-terminal propeptide, whereas the hsPRO-C2 assay described in this study only recognizes the exon 1/3 junction in PIIBNP. The PIIANP levels in OA patients are reduced in comparison to normal subjects [23,27]. Patients with knee OA that have higher baseline PIIANP concentrations demonstrate a greater risk for OA progression [5,32]. Of note, the PIIANP assay is not compatible with plasma. Serum PIICP values indicate type II collagen formation in normal and OA cartilage [33]. The synovial levels of PIICP are increased in OA patients in comparison to healthy individuals [34] and correlate with the BMI [34] and the degree of joint space narrowing [35]. Patients with hypertrophic hip OA show higher serum PIICP levels than patients with atrophic hip OA [36]. Overall, it is unlikely that any single biomarker can offer sufficient sensitivity and specificity to detect early stages of OA, monitor the progress of destruction, accurately and quickly assess the efficacy of therapy, and predict the progression of OA. Thus, there is a need for different types of biochemical markers for different usages in OA. We believe that hsPRO-C2 would be a promising complementary biomarker to the existing formation marker portfolios.

However, some limitations of our study should be noted. First, the clinical cohort is cross-sectional and retrospective in nature, therefore we plan to validate hsPRO-C2 in a prospective study where OA patients are given anabolic treatment. Second, we have not investigated the levels of PIIBNP in the synovial fluid of knee joints from OA patients. Therefore, the circulating PIIBNP in serum likely reflects the whole body cartilage metabolism rather than the local knee joints. Third, PIIBNP may not be specific to knee joints. Although type II collagen is not only the most abundant organic component of articular cartilage but also highly specific for this tissue, it is reported to be expressed in the endochondral bone growth plate and the annulus and nucleus pulposus of intervertebral discs [37,38]. Lastly, the exact structure of PIIBNP in human serum and synovial fluid was not fully explored yet.

To our knowledge, we put forward a serological PIIBNP electrochemiluminescence immunoassay for the first time and investigated its value to reflect systemic type IIB collagen synthesis in human articular cartilage explant treated with IGF-1 and sprifermin. The main findings were as follows: (1) PIIBNP levels had no association with PIIANP levels in pediatric, healthy adult, OA, and RA serum samples. (2) There was a strong response of PIIBNP but a limited response of PIIANP to the putative chondro-anabolic molecules IGF-1 and sprifermin, suggesting that PIIBNP could indicate a synthesis of new type II collagen as a response to therapy. It may indirectly reflect the cartilage formation considering that type II collagen is the major component of articular cartilage. (3) hsPRO-C2 is able to distinguish OA patients from healthy controls. Taken together, the data combined suggest that PIIBNP may be related to different pathologies involving type II collagen formation. To validate these indications, more studies, particularly human longitudinal studies, are needed for further evaluation of the potential of the hsPRO-C2 marker in degenerative joint diseases. This marker may provide additional insight into type II collagen metabolism.

## 4. Methods

### 4.1. Materials

All chemicals were bought from either Sigma-Aldrich (Copenhagen, Denmark) or Merck (Hellerup, Denmark) unless otherwise stated. The synthetic peptides, including biotin-labeled peptide and immunogenic peptide, were obtained from Scilight Peptide Company (Beijing, China). Full-range, rainbow molecular weight markers were bought from GE healthcare (Broendby, Denmark). The monoclonal antibody, NB443-3-2-1 recognizing the N-terminal epitope of PIIBNP, was developed and purified by Protein G column at Nordic Bioscience [28]. 96-well Gold streptavidin microtitre plates, 4× read buffer T with a surfactant, Sulfo-TAG-labeled goat-anti-mouse IgG, and QuickPlex SQ 120 reader with Discovery Workbench software were obtained from Meso Scale Diagnostics (Rockville, MD, USA). The human recombinant PIIANP and PIIBNP were provided by Genscript (Piscataway, NJ, USA). Detailed cloning strategy was shown in Supplementary Figure S1.

Ethical approval for this work was sought and obtained from the local ethics committee at Gentofte Hospital, Gentofte, Denmark and the ethics committee of Northern Jutland, Denmark. The collection of sera samples from patients and healthy subjects was done with informed consent. Sample collections and use were conducted in accordance with the Declaration of Helsinki. The study with 145 OA patients and healthy subjects was approved by The Ethical Committee of Northern Jutland (VEK no.: N-20100094, approval date: 30 November 2015).

### 4.2. Human PIIBNP Extraction from Articular Cartilage

PIIBNP extraction was performed by a modified method as previously described [39]. In brief, normal human articular cartilage from the knee of a 70-year-old Caucasian male (BioIVT, West Sussex, UK) was cut into small pieces. After washing with PBS three times and snap-freezing in liquid nitrogen, the cartilage was ground to coarse particles by using a tissue pulverizer. 632 mg ground cartilage was incubated with 8 mL of 1.4 μg/mL Pronase E solution for 1 h at 37 °C. The supernatant was isolated from cartilage pellets by centrifuging at 1500× *g* for 10 min prior to stopping the enzymatic reaction by adding EDTA to 5 μM. The extracted sample was stored at −20 °C for Western blot use.

### 4.3. Western Blot

The extracted sample above, the recombinant PIIBNP (as a positive control), and recombinant PIIANP were heated at 70 °C for 10 min. In addition, pools of randomly selected serum from three healthy adults, three patients with OA and three patients with RA, the pool of randomly selected synovial fluids of two knee OA patients, and another pool of human amniotic fluid were diluted 5 times in PBS and were heated at 70 °C for 7 min. All the samples were resolved on a 4–12% Bis-Tris gradient gel under reducing conditions. After transferring the proteins from the polyacrylamide gel to a nitrocellulose membrane, the membrane was blocked in Tris-buffered saline (TBS) with 5% skim milk powder and 0.1% Tween-20 for two hours at room temperature (RT) with shaking. The HRP-conjugated monoclonal antibody, HRP-NB443-3-2-1 or HRP conjugated normal mouse IgG1 (negative control), were applied at 1 μg/mL in TBS + 0.1% Tween-20 and 5% skim milk powder overnight at 4 °C with shaking. After further washing, the membrane was detected using enhanced chemiluminescence (ECL) Western blotting substrate (GE healthcare, Denmark). The bands were visualized through exposure to X-ray film.

### 4.4. Histology and Immunohistochemistry

Normal human articular cartilage was mentioned above. Diseased human cartilage was obtained from OA patients who underwent total knee replacement in Gentofte Hospital (Gentofte, Denmark). The tissues were fixed with 4% paraformaldehyde, embedded in paraffin, and cut in five μm sections. The sections were melted for 1 h at 60 °C, followed by deparaffinization in toluene, blocking of endogenous peroxidase activity in 1.02% hydrogen peroxidase (in 99% ethanol), and rehydration through graded ethanol and tap water. Safranin O/fast green was carried out to detect the cartilage. To identify the location of PIIBNP, immunohistochemical staining was carried out in the sections. The tissues were incubated with 2 mg/mL Pronase E (in TBS, pH7.5) for 13 min at 37 °C so as to retrieve the antigen. Following rinsing with TBS + 0.1% Triton X-100, the tissues were blocked with TBS + 0.5% casein for 30 min at RT and then incubated overnight at 4 °C with 10 μg/mL anti-PIIBNP antibody (diluted in TBS + 0.5% casein) or with 10 μg/mL mouse IgG1 isotype control (DAKO, Glostrup, Denmark). After incubation with super enhancer and polymer HRP (BioGenex, Fremont, USA), immunoreactivities were detected using 3,3′-diaminobenzidine (DAB). Mayer’s hematoxylin was used as the counterstain. After each incubation step, the sections were rinsed twice in TBS + 0.1% Triton X-100 for 5 min in each wash. Pictures were captured with a digital camera DP71 (Olympus, Tokyo, Japan) coupled to a BX60 microscope (Olympus, Tokyo, Japan) using 4× or 20× magnification. Image acquisition was achieved using cellSens Entry Software (version 1.15, Olympus, Tokyo, Japan).

### 4.5. Assay Protocol

The hsPRO-C2 competitive ECLIA was developed using electrochemiluminescence (ECL) technology on the MSD platform [40,41]. Such system shows higher sensitivity, low background, minimal matrix effect, and broad dynamic range compared to the traditional ELISA technology [42,43]. Briefly, an MSD Gold 96-well Streptavidin Plate was blocked with 100 μL of blocking buffer (10 mM PBS, 5% BSA, pH 7.4) and incubated for 1 h at 20 °C. After 3 times washing, the plate was coated with 2 ng/mL of the biotinylated synthetic peptide, biotin-QDVRQPG, dissolved in assay buffer (100 mM PBS, 1% BSA, 0.1% Tween-20, 8g/L NaCl, pH 7.4) and incubated for 1 h at 20 °C. The plate was washed 3 times, and 25 μL of the peptide calibrator or sample was added to appropriate wells, followed by 25 μL of 3.9 ng/mL NB443-3-2-1 antibody dissolved in assay buffer. The plate was incubated overnight at 4 °C. Then, 25 μL of 2 ug/mL Sulfo-TAG-labeled goat-anti-mouse IgG was added, and the plate was incubated for 40 minutes at 20 °C. Finally, 150 μL 2× Read buffer was added into the plate before reading on a MESO QuickPlex SQ 120 reader. All the above incubation steps included shaking at 300 rpm. After each incubation step, the plate was washed three times with 10 mM PBS + 0.05% Tween-20, pH 7.4.

### 4.6. Technical Evaluation

Antibody specificity was calculated as a B/B0 percentage of the immunogenic peptide (QDVRQPGPKG), human PIIBNP, and PIIANP recombinant proteins. The lower limit of detection (LLOD) was determined by the MSD software on each analyzed plate. The intra-assay coefficient of variation (intra-CV %) and inter-assay coefficient of variation (inter-CV %) was calculated as the mean value of the variation of five samples analyzed 10 times in duplicate. The measurement range was defined as the range between LLOQ (lower limit of quantification) and ULOD (upper limit of detection, three standard deviations below the mean value of back-calculated Standard A). Linearity dilution and spiking recovery were performed to identify the matrix effect of the serum sample. The analyte stability was determined by three human serum samples for four freeze/thaw cycles and calculated as the percentage recovery of the first cycle. The analyte stability was further tested by incubating three human serum samples for 2, 4, 24, and 48 h at 4 and 20 °C and tested against non-stressed samples.

Matched serum and plasma were obtained via commercial sources (Lee Biosolutions, Inc., St. Louis, MO, USA) from 18 healthy individuals (mean age: 32 ± 9 years) and selected for correlation of hsPRO-C2 levels in plasma and serum.

### 4.7. Comparison of hsPRO-C2 ECLIA and PIIANP ELISA

#### 4.7.1. PIIANP and PIIBNP Profiling in Human OA Cartilage Explants Ex Vivo Model

The HEX culture was performed as described before [44]. Briefly, human OA cartilage was obtained from three OA patients who had a knee replacement surgery in Gentofte Hospital, Denmark. A dermal biopsy punch (diameter: 3 mm, height: 2 mm) was pushed through the cartilage all the way down to the bone, and the cartilage was removed by running the scalpel along the bone surface. The cartilage explants were incubated in DMEM/F-12 (ThermoFisher, Waltham, MA, USA) medium with 1% streptavidin and penicillin in 96-well plate at 37 °C, 5% CO_2_ incubator for ten weeks. The explants were divided into three groups: (1) anabolic stimulation with 900 ng/mL sprifermin; (2) anabolic stimulation with 100 ng/mL IGF-1; (3) DMEM/F-12 (vehicle). There were six replicates for each treatment, two of which were from the same patient. The culture medium was refreshed each week, and the supernatant was stored at −20 °C until measuring type II collagen formation marker (PIIANP and PIIBNP) and degradation marker, C2M (Nordic Bioscience, Herlev, Denmark). The C2M ELISA detects an MMP-9 derived fragment of type II collagen, where a neo-epitope GPPGRDGAAGY at position 1044-1053 was recognized. It has been reported that the C2M level is highly increased in OA patients as well as pro-inflammatory, cytokines-treated articular cartilage explants compared to controls [44]. The metabolic viability of explants was evaluated by the alamarBlue assay (ThermoFisher) at the initiation and termination of the culture. The chondrocytes in the HEX model were encased by the native extracellular matrix, where the cell phenotype could be maintained when culturing for long-term usage. To confirm if the cells are not dedifferentiated into fibroblasts, the collagen type I levels at different time points were measured.

#### 4.7.2. Healthy Subjects and Knee OA Patients Population

A total of 145 participants with knee joint status ranging from no joint degeneration to severe joint degeneration (Kellgren-Lawrence (KL) grades 0–4) were enrolled from Frederikshavn Hospital (Frederikshavn, Denmark). All subjects were Caucasian. Western Ontario & McMaster Universities Osteoarthritis Index (WOMAC) scores and KL grades were recorded. Blood and urine were collected upon overnight fasting prior to surgery or during the consultation. Serum samples were assayed with hsPRO-C2 and PIIANP, respectively.

#### 4.7.3. Correlation of PIIANP and PIIBNP in Human Sera

To test for uniqueness, 10 randomly selected healthy pediatric serum samples (6 female and 4 male; mean age: 10 years), 62 human adult serum samples (11 female and 51 male; mean age: 36.0 years), 155 knee OA (87 female and 68 male; mean age: 64.7 years), and 18 rheumatoid arthritis (RA) serum samples (13 female and 5 male; mean age: 59.4 years) were assessed in the hsPRO-C2 ECLIA and in the PIIANP ELISA (Merck-Millipore/LINCO Research, Burlington, MA, USA). All of the samples were purchased from Lee Biosolutions, Inc. (St. Louis, MO, USA). The OA serum samples were retrieved from patients with Kellgren-Lawrence scores ranging from 2–4. The PIIANP assay is a competitive ELISA based on a polyclonal antibody raised against recombinant GST-human type II procollagen 2 fusion protein [45], which is specific for exon 2 of PIIANP. The intra-assay coefficients of variation (CV) and the inter-assay CV were 4.5 and 6.7% respectively. The lower limit of detection was 30.0 ng/mL. The analyses were performed according to the manufacturer’s instructions [27]. Samples falling above the ULOD were re-assayed at greater dilutions. Undiluted samples falling below LLOQ were imputed by being assigned half the LLOQ as its measured concentration.

### 4.8. Statistical Analysis

All the statistical analysis was carried out with GraphPad Prism (version 7.01) except for the adjustment of body mass index (BMI), sex, and age by MedCal (version 15). The HEX data were normalized to the baseline mean, reported as fold-change of the vehicle prior to ordinary two-way or one-way analysis of variance (ANOVA) with multiple comparisons test. Differences between mean values in the OA cohort were investigated by the non-parametric test (Mann-Whitney). Significance levels were indicated with symbols in the figures where * *p* < 0.05. All values were presented as means and SEMs if not otherwise stated.

## Figures and Tables

**Table 1:** Summary of technical performance for three biomarker assays.

Parameters	PRO-C2 Competition ELISA	hsPRO-C2 Competition ECLIA	PIIANP Competition ELISA
Linear range of standard (ng/mL)	2.5–40	1.0–38.5	32–2000
LLOD (ng/mL)	1.0	0.13	30
Intra-assay % CV (*n* = 10, 10 replicates)	9.2	5.4	4.5
Inter-assay % CV (*n* = 10, 10 replicates)	10.8	5.5	6.7
Analyte stability %, Recovery (*n* = 2, 4× FT, RT 24 h, 2–8 °C 24 h)	110	104	100
Spiking Recovery, % range	102 (74–118)	98 (82–123)	101
Dilution Recovery, % range (*n* = 5)	94 (87–100)	99 (95–102)	102

LLOD: lower limit of detection; CV: coefficient of variation; ELISA: enzyme-linked immunosorbent assay; ECLIA: electrochemiluminescence immunoassay; PIIANP: type IIA collagen NH_2_-propeptide; FT: freeze-thaw; RT: room temperature.

**Table 2:** Demographics of an osteoarthritis cohort. Mann-Whitney test was used to compare the difference of clinical parameters between the groups. * *p* < 0.05, compared to control group. ** *p* < 0.01, compared to control group. KL: Kellgren and Lawrence, VAS: visual analog scale, BMI: body mass index.

KL Grade	*Number*	Female %	Age (Mean, 95% CI)	VAS (Mean, 95% CI)	BMI (Mean, 95% CI)
0, 1 (Control)	22	40.9	62.2 (58.3–66.1)	32.5 (21.3–43.6)	26.0 (24.6–27.5)
2, 3, 4 (OA)	123	56.1	65.2 (63.9–66.5)	49.4 (45.0–53.8) *	28.3 (27.6–28.9) **

**Table 3:** Correlation (r) between hsPRO-C2 and PIIANP serum levels in randomly selected pediatric, healthy adults, OA, and RA samples. Results are Pearson’s correlation coefficients, adjusted for age, body mass index and gender. ns = non-significant difference.

Samples	*R*	Number	*p* Value
Pediatric serum	<0.1	18	0.7863 (ns)
Healthy adult serum	−0.146	62	0.1857 (ns)
OA serum	0.148	155	0.0648 (ns)
RA serum	<0.1	10	0.7524 (ns)

## Supplementary Materials

The following are available online at http://www.mdpi.com/1422-0067/19/11/3485/s1.

## Abbreviations

BMI	Body mass index
DMOAD	Disease-modifying osteoarthritis drug
ECLIA	Electrochemiluminescence immunoassay
ECM	Extracellular matrix
IGF-1	Insulin-like growth factor 1
LLOD	Lower limit of detection
LLOQ	Lower limit of quantification
MRI	Magnetic resonance imaging
OA	Osteoarthritis
PBS	Phosphate buffered saline
PINP	Procollagen type I N-terminal propeptides
PIIANP	Procollagen type IIA N-terminal propeptides
PIIBNP	Procollagen type IIB N-terminal propeptides
RA	Rheumatoid arthritis
SD	Standard deviation
SDS-PAGE	Sodium dodecyl sulfate-polyacrylamide gel electrophoresis
ULOD	Upper limit of detection
