# Supplementary material for: A Novel High Sensitivity Type II Collagen Blood-Based Biomarker, PRO-C2, for Assessment of Cartilage Formation
Source: Int J Mol Sci. 2018 Nov 6;19(11):3485. doi: 10.3390/ijms19113485 (PMC6275061; doi:10.3390/ijms19113485)
Supplement: Supplementary file 1 [file ijms-19-03485-s001.pdf]

Supplementary data

SEQ1: PIIANP

EcoRI--Kozak sequence--Artificial signal peptide--His6 tag--TEV--Human COL2A1 (26~181)--stop codon--HindIII

Protein Sequence: 188 aa

MGWSCIILFLVATATGVHSHHHHHHENLYFQGQDVQEAGSCVQDGQRYNDKDVWK-
PEPCRICVCDTGTVLCDDIICEDVKDCLSPEIPFGECCPICPTDLATASGQPGPKGQKGEPGDIKDIVGPKGPPGPQ
GPAGEQGPRGDRGDKGEKGAPGPRGRDGEPGTPGNPGPPGPPGPPGPPGLGGNFAA.

DNA sequence: 591 bp

GAATTCCCGCCGCCACCATGGGCTGGTCCTGCATCATTCTGTTTCTGGTGGCCACAGCCAC-
CGGCGTGCACTCTCACCATCACCACCATCATGAGAACCTGTACTTCCAAGGCCAGGACGTGCAAGAGGCCGGA
AGCTGTGTTCAGGACGGCCAGAGATACAACGACAAGGACGTGTGGAAGCCCGAGCCTT-
GCAGAATCTGCGTGTGTGATACCGGCACCGTGCTGTGCGACGACATCATCTGCGAGGACGTGAAGGACTGTC
TGAGCCCTGAGATCCCTTTCGGCGAGTGCTGCCCTATCTGCCCTACAGATCTGGC-
TACAGCCTCTGGCCAGCCTGGACCTAAAGGACAGAAAGGCGAGCCCGGCGACATCAAGGATATCGTGGGACC
TAAGGGCCCTCCTGGACCACAAGGACCTGCTGGCGAACAAGGACCCAGAGGCGATA-
GAGGCGACAAGGGCGAAAAAGGCGCTCCAGGACCTAGAGGCAGAGATGGCGAACCTGGCACACCTGGCAAT
CCAGGACCACCTGGTCCTCCAGGTCCTCCTGGGCCACCTGGACTCGGAGGAAATTTTGCTGCTTGATAAGCTT

SEQ 2: PIIBNP

EcoRI--Kozak sequence--Artificial signal peptide--His6 tag--TEV--Human COL2A1 (26~112)--stop codon--HindIII

Protein Sequence: 119 aa

MGWSCIILFLVATATGVHSHHHHHHENLYFQGQDVRQPGPKGQKGEPGDIK-
DIVGPKGPPGPQGPAGEQGPRGDRGDKGEKGAPGPRGRDGEPGTPGNPGPPGPPGPPGPPGLGGNFAA.

DNA sequence: 384 bp

GAATTCCCGCCGCCACCATGGGCTGGTCCTGCATCATTCTGTTTCTGGTGGCCACAGCCAC-
CGGCGTGCACTCTCACCATCACCACCATCATGAGAACCTGTACTTCCAAGGCCAGGACGTGCGACAGCCTGGA
CCTAAAGGACAGAAAGGCGAGCCCGGCGACATCAAGGATATCGTGGGAC-
CTAAGGGCCCTCCTGGACCACAAGGACCTGCTGGCGAACAAGGACCCAGAGGCGATAGAGGCGACAAGGGC
GAAAAAGGCGCTCCAGGACCTAGAGGCAGAGATGGCGAACCTGGCACACCTGGCAATCCAG-
GACCACCTGGTCCACCTGGACCTCCAGGGCCACCAGGACTCGGAGGAAATTTTGCTGCTTGATAAGCTT

Figure S1. Cloning strategy of recombinant PIIANP and PIIBNP. The complete DNA sequence of human recombinant PIIANP and PIIBNP was sub-cloned into the pcDNA3.4 vector. Detailed cloning strategies were shown in Figure 1. Expi293FTM cells were grown in serum-free Expi293TM Expression Medium (ThermoFisher Scientific). The cells were maintained in Erlenmeyer Flasks (Corning Inc.) at 37°C with 8% CO_2 on an orbital shaker (VWR Scientific). One day before transfection, the cells were seeded at an appropriate density in Corning Erlenmeyer Flasks, where DNA and ExpiFectamine™ 293 Reagent were mixed at an optimal ratio and then added into. The recombinant plasmid encoding target protein was transiently transfected into suspension Expi293F cell cultures. Approximately 16–18 hours post-transfection, ExpiFectamine™ 293 Transfection Enhancer 1 and ExpiFectamine™ 293 Transfection Enhancer 2 were added to each flask. The cell culture supernatants collected on day 6 were used for affinity purification by HisTrapTM FF Crude. The his tags will be removed by treating the purified recombinant proteins with TEV protease.

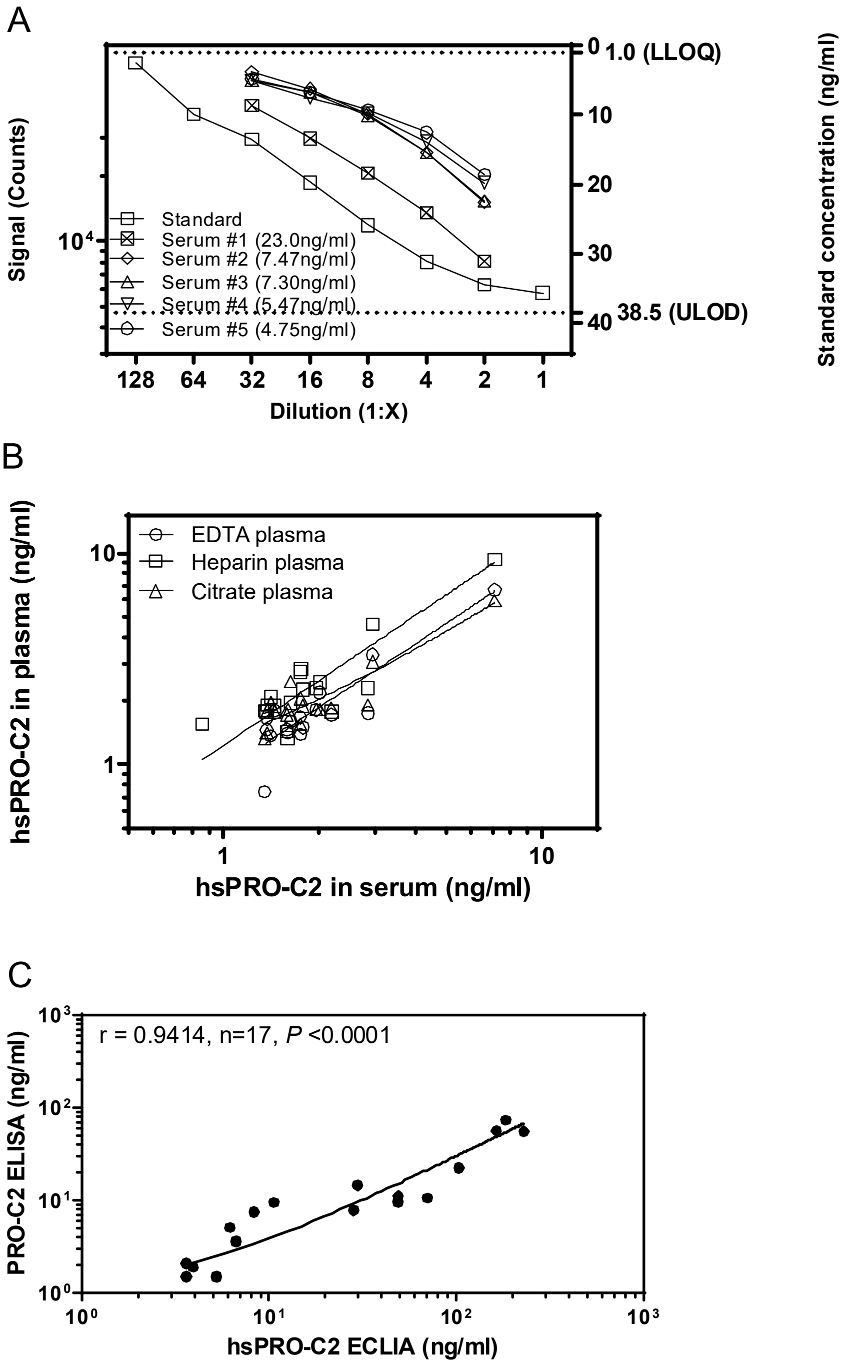

Figure S2. (A) Dilution curves of PIIBNP standard and samples. Serum concentrations from different subjects analyzed in the hsPRO-C2 ECLIA assay plotted against the dilution. Sample concentrations of PIIBNP at different dilutions were calculated against the standard curves using four-parameter logistics. Dilutions falling within the range of detection as defined by the Upper and Lower Limit of Quantification (ULOD and LLOQ; described in the Methods section) were used for linearity of dilution calculations (Table 1), with mean values

presented in the legends. (B) Correlation of PIIBNP levels in three kinds of plasma and serum (matched samples, from 18 individuals). Linear regression analysis was performed, plasma vs serum. The figure shows the strong correlations of PIIBNP levels in serum and plasma (P < 0.0001). (C) Correlation of hsPro-C2 ECLIA and PRO-C2 ELISA in 17 randomly selected bovine explants supernatant samples. Pearson's correlation coefficient (r), and a regression line (solid) are indicated.

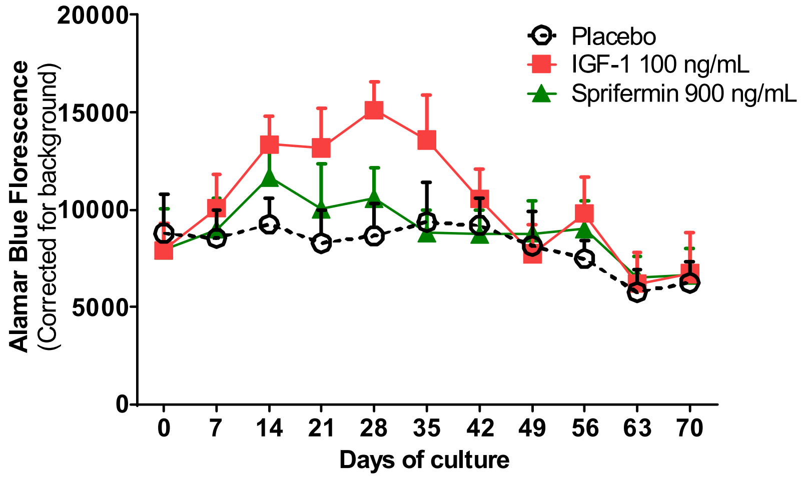

Figure S3. Metabolic activity of human articular cartilage explants by measuring alamarBlue. No significant differences were seen among the groups.
